# An unexpected diagnosis of human immunodeficiency virus-2 infection in an overseas visitor: a case report

**DOI:** 10.1186/s13104-017-2438-7

**Published:** 2017-03-04

**Authors:** Asma Sohail, Lyndal Van Leer, Natasha Holmes

**Affiliations:** 1 Department of Infectious Diseases, Austin Health, 145 Studley Road, Heidelberg, VIC Australia; 2 Department of General Medicine, Austin Health, Heidelberg, VIC Australia; 3 Department of Microbiology and Immunology, University of Melbourne, Melbourne, VIC Australia

**Keywords:** Human immunodeficiency virus-2, HIV-2, Antiretroviral therapy, Diagnostic testing

## Abstract

**Background:**

Human immunodeficiency virus 2 infection is endemic in West Africa but is also found in parts of Europe, North and South America, and India where it is thought to have been introduced secondary to migration and commercial trade ties. It is less common than Human immunodeficiency virus 1, with differences in pathogenicity, lower rates of transmission, longer asymptomatic period and slower progression to acquired immunodeficiency syndrome. Human immunodeficiency virus 2 is also associated with diagnostic challenges given the lack of commercially available diagnostic tests, and management challenges given intrinsic resistance to many anti-retroviral therapies.

**Case presentation:**

We describe a case of a 65 year old South Indian female, visiting her family in Australia, who presented with weight loss, pancytopaenia and generalised lymphadenopathy on a background of newly diagnosed congestive cardiac failure. Multiple investigations were performed to elucidate the cause of her presentation, with the eventual unexpected diagnosis of human immunodeficiency virus 2. She was commenced on anti-retroviral treatment and made a remarkable recovery.

**Conclusion:**

We describe the challenges associated with diagnosis of human immunodeficiency virus 2 due to lack of commercially available diagnostics, as well as the treatment and management challenges including the fact that human immunodeficiency virus 2 is intrinsically resistant to non-nucleoside reverse transcriptase inhibitors. Human immunodeficiency virus 2 infection should be considered in patients who present with symptoms and signs that do not point towards a clear diagnosis, such as unexplained pancytopaenia or lymphadenopathy, and who have risk factors such as being from an endemic area or having had blood transfusions, especially prior to the commencement of blood-borne virus screening of blood donors.

## Background

The World Health Organisation estimates that 36.9 million people are currently living with human immunodeficiency virus [1], of which approximately 1–2 million are thought to be infected with human immunodeficiency virus 2 (HIV-2). HIV-2 is less pathogenic than human immunodeficiency virus 1 (HIV-1) and the majority of HIV-2 infected individuals are long-term non-progressors [2]. The transmission rate is thought to be much lower and there is a longer period of asymptomatic infection with a slower decline in CD4 cell counts [2, 3]. This ultimately means that there is a slower progression to acquired immunodeficiency syndrome (AIDS) and mortality in HIV-2 infection is also more favourable compared to HIV-1 [2, 3]. Diagnosis can be challenging due to the lack of commercially available diagnostic tests. Moreover, management can be difficult due to HIV-2 being intrinsically resistance to several classes of antiretroviral medications and due to the lack of commercially available genotype and resistance assays and viral load assays.

## Case presentation

Mrs. M.R, a 65-year-old South Indian woman, visiting family in Australia, was admitted in January 2015 for the management of new congestive cardiac failure (CCF).

She had been recently discharged from a different hospital with symptoms consistent with community-acquired pneumonia and managed with intravenous ceftriaxone and azithromycin followed by amoxicillin/clavulanate and roxithromycin. Investigations at this time included a chest X-ray demonstrating right lung lower zone consolidation, and blood and sputum cultures were negative for routine pathogens; sputum acid-fast bacilli (AFB) smears were negative. She subsequently developed worsening bilateral peripheral limb oedema, increasing exertional dyspnoea and paroxysmal nocturnal dyspnoea, and was admitted to our hospital for further management. Clinical examination was consistent with congestive cardiac failure with evidence of atrial fibrillation. Additionally the patient was found to have cervical and parotid lymphadenopathy measuring approximately 1–2 cm in size.

Her past medical history included ischaemic heart disease, atrial fibrillation, hypertension, osteoarthritis, asthma, herpes zoster (6 months prior to admission), laparotomy in 1968 for an unclear indication, and total hysterectomy in 1991. Further history revealed a 6-month history of 5 kg weight loss with no anorexia, and she denied fevers and night sweats. There was no other travel history besides to Australia. She had been married since the age of 19 and denied other sexual partners. There was no history of tattoos or injecting drug use. She had no animal contact or gardening activities.

Initial laboratory investigations revealed pancytopenia with a normocytic anaemia: haemoglobin 80 g/L (115–165 g/L), mean cell volume 84 fL (80–96 fL), white cell count 3.0 × 10^9^/L (4–11 × 10^9^/L), with normal neutrophils 2.5 × 10^9^/L (2–7.5 × 10^9^/L) and lymphopenia 0.2 × 10^9^/L (1–4 × 10^9^/L), and platelets 55 × 10^9^/L (150–400 × 10^9^/L). Her chest X-ray was consistent with fluid overload with bilateral pleural effusions and there was evidence of right lung lower zone consolidation.

Given the pancytopenia, lymphadenopathy and weight loss, Mrs. M.R proceeded to have computerised tomography (CT) of the neck, chest, abdomen and pelvis. This revealed widespread mediastinal, hilar and peritoneal lymphadenopathy as well as multiple peripherally enhancing cystic lesions within the right parotid gland, raising the possibility of tuberculous adenitis. A positron emission tomography scan demonstrated metabolically active retroperitoneal, mesenteric and inguinal lymph nodes. Transthoracic echocardiography revealed normal left ventricular size and function, impaired right ventricular systolic function as well as diastolic dysfunction, and severe pulmonary hypertension (pulmonary artery systolic pressure 57 mmHg + right atrial pressure). CT pulmonary angiogram did not reveal evidence of pulmonary embolus, and screening tests for connective tissue disorders as a cause for secondary pulmonary hypertension were unremarkable. In view of her country of origin and respiratory symptoms, sputum AFB smears were repeated and were negative. Quantiferon-Gold testing was indeterminate.

**Fig. 1:**
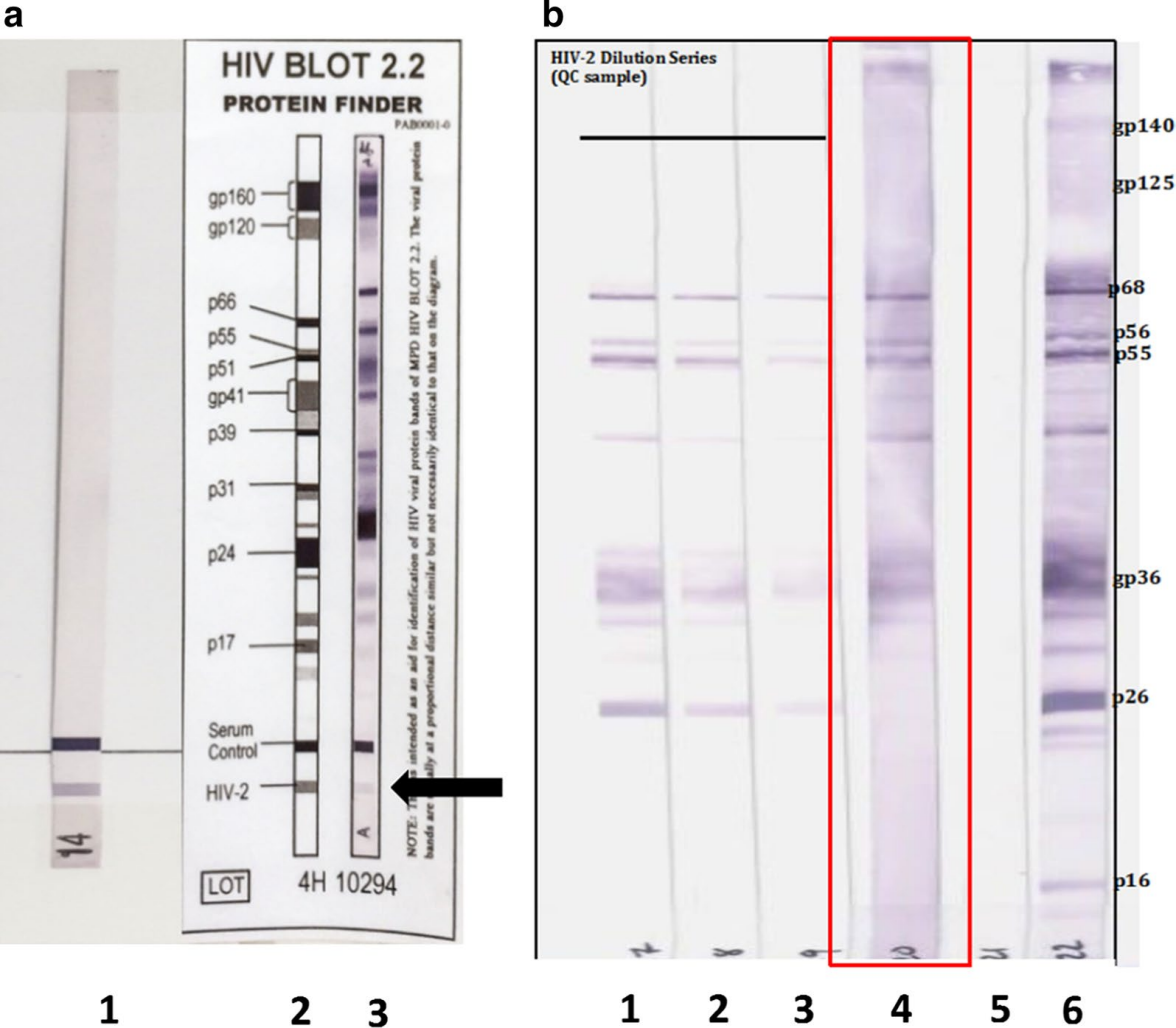
Mrs M.R’s HIV-1 and HIV-2 western blot assays. **a** HIV-1 western blot at the Victorian Infectious Diseases Reference Laboratory. *Lane 1* control sample; *lane 2* interpretation schema; *lane 3* patient sample. *Black arrow* indicates positive band corresponding to HIV-2. Multiple bands detected in *lane 3* do not match HIV-1 proteins. **b** HIV-2 specific western blot at the National Reference Laboratory. *Lane 1* quality control (QC) 1:2048 dilution; *lane 2* QC 1:4096 dilution; *lane 3* QC 1:8192 dilution; *lane 4* patient sample (highlighted in *red*); *lane 5* negative control; *lane 6* positive control with interpretation schema. Note patient has multiple HIV-2 bands matching QC and positive control samples

Given her unexplained lymphadenopathy and weight loss, Mrs. M.R went on to have human immunodeficiency virus testing. The HIV-1/2 enzyme immunosorbent assay (EIA) (Roche) at our hospital was unexpectedly strongly positive on two occasions and the sample was sent to the Victorian Infectious Diseases Reference Laboratory for confirmation. The HIV-1/2 EIA using different platforms (Genscreen and Liaison XL) was also positive but the HIV-1 p24 antigen was negative. The HIV-1 western blot was negative. This prompted testing for HIV-2, performed at the National Reference Laboratory, using an in-house HIV-2 western blot (Fig. 1) and the BioRad Multispot EIA assay, which is able to differentiate between HIV-1 and HIV-2 antibodies. Both tests confirmed HIV-2 infection. HIV-2 viral load (VL) was performed using a research-based assay and was detected at 3260 copies/mL. Resistance testing demonstrated susceptibility to all protease inhibitors and nucleoside reverse transcriptase inhibitors; resistance to non-nucleoside reverse transcriptase inhibitors was consistent with HIV-2. Her CD4 T cell count was 118 cells/µL (17%) (650–2000 cells/µL; 35–59%). Further questioning revealed the patient received a blood transfusion in 1991 during her hysterectomy in India; no other risk factors for HIV infection were determined, and her husband tested negative for HIV-1 and HIV-2.

Given this new diagnosis of HIV-2 infection, a number of potential differential diagnoses were considered for Mrs. M.R’s pancytopenia, widespread lymphadenopathy, weight loss and ongoing respiratory issues. Multiple lymph node aspirates and core biopsies, bone marrow aspirate and trephine, and bronchoscopy were performed and did not reveal a microbiological or haematological diagnosis; investigations for mycobacterial, fungal and opportunistic infections were negative on all specimens.

She was commenced on anti-retroviral therapy with fixed-dose combination emtricitabine 200 mg + tenofovir 300 mg daily and ritonavir (200 mg)-boosted lopinavir 800 mg daily, a regimen widely available in India. She was also commenced on trimethoprim-sulfamethoxazole for *Pneumocystis jiroveci* prophylaxis. Within 2 months her VL was undetectable and her CD4 cell count was improving, after an initial decline. Her pancytopenia improved and her lymphadenopathy regressed over the next 2 months and these were subsequently attributed to HIV-2 infection.

Mrs. M.R continued on anti-retroviral treatment and remained well until she returned to India.

## Discussion

The World Health Organisation estimates that 36.9 million people are currently living with HIV [1], of which approximately 1–2 million are thought to be infected with HIV-2. HIV-2 was first isolated in humans in Senegal in 1984 [4] and originated from sooty mangabeys. It is endemic in West Africa but is also found in parts of Europe, North and South America, and India where it is thought to have been introduced secondary to migration and commercial trade ties [2]. Its prevalence is very rare in Australia [5].

Phylogenetic analysis confirms that HIV-1 and -2 are genetically distinct [4]. HIV-2 is less pathogenic than HIV-1 and the majority of HIV-2 infected individuals are long-term non-progressors [2]. Although transmission routes for HIV-2 are the same as for HIV-1, the transmission rate is thought to be much lower, with heterosexual transmission rates 5- to 10-fold lower [6] and vertical transmission rates 6- to 30-fold lower [2] compared with HIV-1 infection. There is a longer period of asymptomatic infection and slower decline in CD4 cell counts, thought to be secondary to lower levels of plasma viraemia [3]. This ultimately means that there is a slower progression to acquired immunodeficiency syndrome and only 20–30% of HIV-2 infected individuals go on to develop AIDS [2, 3]. Mortality in HIV-2 infection is also more favourable compared to HIV-1 at CD4 counts above 200 cells/μL, however this advantage is lost when CD4 counts drop below 200 cells/μL [2].

HIV diagnosis and management guidelines from the US [7] recommend testing for HIV-2 in specific patient populations (Table 1). The differentiation between HIV-1 and HIV-2 infection is important given the differing natural histories of the disease and subsequent choice of anti-retroviral therapy (ART). The HIV-1/HIV-2 type-differentiation assay is only available in a research setting in Australia and assisted in the diagnosis in our patient. A western blot is performed for confirmation of HIV-2. Unfortunately, HIV-1 western blots may fully or partially cross react with HIV-2, giving positive, indeterminate or negative results that could lead to an incorrect diagnosis of HIV-1 or a missed diagnosis of HIV-2. In-house assays for HIV-2 viral load in the US and Australia have not been approved by regulatory authorities. Therefore, monitoring of treatment response in HIV-2 is difficult due to the lack of commercially available viral load monitoring and genotypic resistance assays.

**Table 1:** Differentiation of HIV-1 and HIV-2, based on the *New York State Department of Health AIDS Institute*

1	Originated or have travelled to HIV-2 endemic area
2	Received medical care, injections, immunisations, phlebotomy, surgery or blood products in an HIV-2 endemic area
3	Sexual or needle sharing contact with persons who are infected with HIV-2 or from a HIV-2 endemic area
4	Born to a mother with HIV-2 infection
5	Opportunistic infections or other clinical symptoms of HIV/AIDS but tested negative or indeterminate for HIV-1
6	Multiple HIV-1 indeterminate antibody test results
7	Unusual pattern/indeterminate HIV-1 Western Blot
8	Confirmed diagnosis of HIV-1 but undetectable viral load incompatible with clinical or immunological status

http://www.hivguidelines.org/clinical-guidelines/adults/human-immunodeficiency-virus-type-2-hiv-2/

The goals of therapy in HIV-2 are similar to HIV-1, however the choice of anti-retroviral therapy differs for HIV-2. There are no randomised controlled trials to assess the efficacy of specific combination ART in treatment-naïve HIV-2 infected patients.

HIV 2 is intrinsically resistant to non-nucleoside reverse transcriptase inhibitors (NNRTIs) and to the fusion inhibitor enfuvirtide [2]. Both these classes of ART are *not* recommended for treatment of HIV-2 infection. In vitro and small observational studies have shown that nucleoside reverse transcriptase inhibitors (NRTIs) and protease inhibitors (PIs) are active against HIV-2 [2]. Among PIs, lopinavir, saquinavir and darunavir have been shown to be most potent [8]. HIV-2 appears to be susceptible to the integrase strand transfer inhibitors (INSTIs), and recent trials have shown good responses [9]. However, there are less long-term data compared to PIs and emergence of on-treatment mutations have been reported. As HIV-2 can use several co-receptors to enter cells, some with greater affinity than CCR5, the efficacy of the CCR5 antagonist maraviroc is uncertain [2]. Current British and US guidelines suggest starting a NRTI backbone with a boosted PI [10, 11] or INSTI [11], and these are reflected in Australian guidelines [5].

## Conclusion

In conclusion HIV-2 infection was an unexpected diagnosis in Mrs M.R, with the only risk factors identified being a blood transfusion in 1991 and being from India where HIV-2 is not uncommon. We did not find any evidence of an opportunistic infection or lymphoproliferative disorder to explain her lymphadenopathy and cytopenias, and it was assumed these were HIV-related in the context of advanced disease. Investigations to look for other causes of her pulmonary hypertension were also negative and it was thought this was possibly related to advanced HIV-2 infection and pre-existing ischaemic heart disease. Clinicians need to be aware of HIV-2 as a diagnosis and modern algorithms should incorporate testing. Although HIV-2 infection is uncommon, patients have a prolonged asymptomatic phase and may present late due to its lower pathogenicity. HIV-2 viral load and genotypic resistance assays are not commercially available, making testing as well as monitoring treatment response difficult. Management of HIV-2 differs from HIV-1; NNRTIs and fusion inhibitors cannot be used, and current guidelines recommend the use of 2 NRTIs and a boosted PI or INSTI.

This case also highlights the importance of undertaking HIV testing in patients with a constellation of symptoms and signs that do not point towards a clear diagnosis and to consider HIV-2 in patients who come from an endemic area or have other risk factors such as blood transfusions and sexual contact with someone from an endemic area.

## Abbreviations

HIV: human immunodeficiency virus
AIDS: acquired immunodeficiency syndrome
CCF: congestive cardiac failure
AFB: acid fast bacilli
CT: computerized tomography
EIA: enzyme immunosorbent assay
VL: viral load
ART: antiretroviral therapy
NNRTI: non nucleoside reverse transcriptase inhibitor
NRTI: nucleoside reverse transcriptase inhibitor
PI: protease inhibitor
INSTI: integrase strand transfer inhibitor
